# Resilience and well-being of university nursing students in Hong Kong: a cross-sectional study

**DOI:** 10.1186/s12909-018-1119-0

**Published:** 2018-01-12

**Authors:** Ka Ming CHOW, Wing Ki Fiona TANG, Wing Han Carmen CHAN, Wing Hung Janet SIT, Kai Chow CHOI, Sally CHAN

**Affiliations:** 1 The Nethersole School of Nursing, The Chinese University of Hong Kong, Hong Kong SAR, China; 2 School of Nursing and Midwifery, Faculty of Health and Medicine, The University of Newcastle, New South Wales, Australia

**Keywords:** Resilience, Well-being, Nursing students, Nursing education

## Abstract

**Background:**

University nursing students experience higher levels of academic stress than those of other disciplines. Academic stress leads to psychological distress and has detrimental effects on well-being. The ability to overcome such adversity and learn to be stronger from the experience is regarded as resilience. Resilience is found to have an impact on learning experience, academic performance, course completion and, in the longer term, professional practice. Resilience and positive coping strategies can resist stress and improve personal well-being. However, the relationship between resilience and well-being remains unexplored in nursing students, which are significant attributes to their academic success and future career persistence.

**Methods:**

The study was a cross-sectional descriptive correlational design. Inclusion criteria for recruitment was students studying pre-registration nursing programmes at both undergraduate and postgraduate levels. The 10-item Connor-Davidson Resilience Scale (CD-RISC-10) and World Health Organisation-5 Well-Being Index (WHO-5) were used to measure resilience and psychological well-being respectively.

**Results:**

A convenience sample of 678 university nursing students was recruited from a university. The mean score of CD-RISC-10 was 24.0. When comparing the resilience levels of undergraduate and postgraduate students, the total scores were found to be 23.8 and 24.9 respectively. There was a statistically significant difference between the groups (*p* = .020). With regard to perceived well-being, the mean score of WHO-5 was 15.5. There was no significant difference between undergraduates and postgraduates (*p* = .131). Bivariate analysis showed that self-reported resilience had a medium, positive correlation with perceived well-being (*r* = .378, *p* = .000), and senior students had significantly higher level of perceived well-being than junior students (16.0 vs 15.1, *p* = .003). Multivariable regression analysis on perceived well-being indicated that self-reported resilience emerged as a significant predictor of perceived well-being (regression coefficient B = 0.259; *p* < .001).

**Conclusions:**

The results demonstrate that nursing students with a high level of resilience have better perceived well-being, and the level of resilience of postgraduates was significantly higher than that of undergraduates. Therefore, educational strategies should be developed in the nursing curriculum and a supportive learning environment should be created to foster resilience in the students.

## Background

Nursing is becoming ever more demanding, and is regarded as a stressful occupation because of manpower shortages and various other challenges associated with nursing practice [1]. While studying nursing at university, students experience higher levels of academic stress than those of other disciplines as they have to adapt to various clinical settings for practice, and are exposed to diverse patient conditions as well as dying and death during placement [2–4]. Academic stress leads to psychological distress and has detrimental effects on well-being. In addition, nursing students have to apply theoretical knowledge to practice in order to bridge the theory-practice gap [5]. They have to cope with the emotional and academic demands of patient care [3]. All these stressors may increase stress and psychological morbidity, such as excessive anxiety, worry and depression. The ability to overcome such adversity and learn to be stronger from the experience is regarded as resilience, a concept that emerged in the 1970s [5, 6]. Resilience is imperative for nursing students to survive adversity and prepare them for undertaking professional role after graduation [4, 7]. In a conceptual model of resilience in nursing students, resilience is conceptualised as a process of cumulative success in overcoming adversity, which enhances personal well-being [4]. It is known that chronic exposure to stressors contributes to poor well-being, resulting in lower job satisfaction [8], which may well affect nursing students’ aspirations to take up a clinical post after graduation [9].

Previous study has found that resilience has an impact on learning experience, academic performance, course completion and, in the longer term, professional practice [3]. Studies have also investigated whether resilience affects the academic performance of undergraduates in different disciplines. In the case of hospitality and tourism students, resilience is a significant predictor of academic performance [10]. In the aspect of nursing, resilience level of the students varies across countries. Nursing students in Nigeria showed a moderate level of resilience [11], while their counterparts in Australia and Spain reported a high level of resilience [12, 13]. Resilient nursing students showed better psychological health and lower academic burnout [13]. There is a growing research interest in exploring why some nursing students can cope well but others cannot when they all face similar problems and challenges during their programme. Resilient individual nurses would have a tendency to look for positive meanings in negative circumstances, so that they can cope with distress effectively and adopt the knowledge they acquire from the setback as a form of reference to help them cope with similar situations in the future [14]. It is important to note that resilience is not merely an indicator of well-being, but is a process that enables an individual to remain healthy or to recover quickly after adversity [15].

Well-being is a broad subjective concept with three main components: life satisfaction, pleasant affect and unpleasant affect [16, 17]. It has been suggested that being resilient is a necessary building block of well-being. Resilience and positive coping strategies can resist stress and improve personal well-being [18]. Resilient students are therefore taken to have better perceived well-being. As a result, the development of resilience and active coping strategies has been highlighted as critical for success and continuance in the nursing profession. Nursing faculties therefore have a responsibility to help build student well-being through the development of resilience and effective coping skills [3, 19].

Despite the benefits of resilience to health, its relationship with the well-being of nursing students remains unexplored. To our knowledge, no study on resilience and well-being among university nursing students has been conducted in Hong Kong. At the same time, nursing education in Hong Kong is more intense than in other countries, for example Australia and Singapore, in the clinical hours required [20]. The clinical environment is also stressful for health professionals and nursing students in the local context, with a disproportionate nurse to patient ratio of 1:10 or 12 in the daytime and 1:16 up to 20 on night duty [21]. Encountering such stressful working environments, health professionals tend to suppress their negative reactions, as Chinese culture emphasises endurance and the suppression of emotions [22]. As a result, they may experience and perceive resilience differently.

The present study aims to examine the relationship between resilience and well-being among university nursing students in Hong Kong. We hypothesise that nursing students with higher levels of resilience will have higher levels of well-being.

## Methods

### Study design, participants and setting

The study was a cross-sectional descriptive correlational design. Students were recruited from pre-registration nursing programmes at both undergraduate and postgraduate levels at the Chinese University of Hong Kong. Students in these programmes undertake the same curriculum, which is required by the Nursing Council of Hong Kong for registration as registered nurses [20]. However, nursing undergraduates follow a five-year full-time programme, to which they are admitted directly from secondary schools to pursue their first degree. Undergraduate students usually enter the university at age 18, while postgraduates follow a three-year full-time programme which is designed for those with a first degree in other disciplines but who want to pursue a second career. Compared with undergraduate nursing students, postgraduate ones are usually older and their study schedule is more packed. For both programmes, clinical practice is integrated in the curriculum with theoretical inputs. Undergraduate students start their clinical placement in year two of study and the duration gradually increases from four weeks to 18 weeks in the final year. Students with adequate clinical exposure are regarded as senior students. Therefore, year one to three students in undergraduate programme are regarded as junior students, while those studying in year four and five are identified as senior students. Clinical placement of postgraduate students increases from eight weeks in year one to 19 weeks in year three of study. Postgraduate year one nursing students are regarded as junior students, while those studying in the second and final year are senior ones. All eligible students were invited to participate, and data collection was conducted between November 2016 and February 2017.

### Data collection procedure

All eligible students were approached by trained research assistants at the end of their lectures. Information sheets about the study and consent forms were distributed. Students chose whether to take part or not on a voluntary basis and were quite free to decline if unwilling to join for some reason. After written consent was obtained, participants were asked to complete the questionnaires, which took around 15–20 min.

### Outcome measures

#### Connor-Davidson resilience scale (CD-RISC)

The 10-item CD-RISC (CD-RISC-10) was used to measure resilience in university nursing students. It captures core features of resilience over the preceding month. Each item is rated on a five-point Likert scale ranging from 0 ‘never’ to 4 ‘almost always’, with higher scores indicating greater resilience [23]. The scale has been tested in undergraduate samples and student nurses with satisfactory reliability and validity [11, 23].

#### World Health Organisation-5 well-being index (WHO-5)

The WHO-5 is an instrument developed for assessing psychological well-being over a two-week period. The five items in the scale cover positive mood (feeling in good spirits, feeling relaxed), vitality (being active and waking up fresh and rested), and being interested in things. Each item is rated on a six-point Likert scale from 0 ‘at no time’ to 5 ‘all of the time’, with higher scores representing better self-perceived well-being. The total scores range from 0 to 25, with a score below 13 indicating poor well-being and the need to test for depression [24, 25]. The WHO-5 has been translated into more than 30 languages. It has been widely used in research studies and tested in various populations with good psychometric properties [26].

### Statistical data analyses

Data analyses were performed by using IBM SPSS 24.0 (IBM Crop., Armonk, NY). Appropriate descriptive statistics, including frequency (percentage) and mean (standard deviation, SD) were used to present the background characteristics, including gender, study programme and year of study, and self-report resilience and perceived well-being measures of the sample. To compare any difference in resilience and well-being between students in the undergraduate and postgraduate pre-registration programmes, as well as between junior and senior students, independent *t*-tests were conducted to examine the results. Bivariate analyses by means of Pearson correlation and independent *t*-test, as appropriate, were performed to examine the associations between perceived well-being and self-report resilience as well as the background characteristics of university nursing students, including sex, programme of study (undergraduate or postgraduate) and whether in senior year or not. Those factors showing significance (*p* < .05) in bivariate analyses were selected for multivariable regression analysis to delineate factors significantly associated with perceived well-being. All statistical tests were two-sided and the level of significance was set at 0.05.

### Ethical considerations

Ethics approval was obtained from the Survey and Behavioural Research Ethics Committee (SBREC) of the Chinese University of Hong Kong. Participation was voluntary and anonymous, and recruits were given an explanation of the aims and nature of the study and their right to withdraw at any times clarified. In view of the packed time table and data was collected after lectures, HKD20 cash was given to each participant on return of the questionnaire to encourage their participation and compensate for their time. The incentive for participation was approved by the SBREC. An information sheet with details of the study was given to all participants, who were assured that personal information collected would be kept strictly confidential and used solely for this study: electronic data would be stored in a password-protected researcher’s computer, and only the investigating team and their research staff would have access to the data during and after the study. All hard copies of questionnaires would be kept in a locked cabinet, with the researchers responsible for their safekeeping.

## Results

### Participant characteristics

A convenience sample of 678 university nursing students was recruited, 508 females and 170 males, of whom 474 were in the undergraduate (69.9%) and 204 in the postgraduate programme (30.1%). Of these undergraduates, 298 were junior students in years one to three (62.9%) and 176 in senior years four or five (37.1%). Of the postgraduates, 75 were in year one (36.2%) and 129 in years two or three (63.8%). All participant characteristics are summarised in Table 1.

**Table 1:** Background characteristics of the university nursing students (*N* = 678)

Background characteristics	n (%)
*Sex*	
Female	508 (74.9%)
Male	170 (25.1%)
*Programme*	
Undergraduate	474 (69.9%)
Postgraduate	204 (30.1%)
*Senior year student*	
No	373 (55.0%)
Yes	305 (45.0%)

Data of the characteristics are presented as frequency (percentage)

### Self-reported resilience

A summary of university nursing student outcome measures is shown in Table 2. The total scores in CD-RISC-10 ranged from 7.0 to 40.0, with a mean of 24.0 (SD = 5.7). When comparing the resilience levels of undergraduate and postgraduate students, the total scores were found to be 23.8 and 24.9 respectively. There was a statistically significant difference between the groups (*p* = .020). However, no significant difference in resilience level was found between junior and senior students (*p* = .912).

**Table 2:** Self-report resilience (CD-RISC-10 total score) and perceived well-being (WHO-5 total score) of the university nursing students

	CD-RISC-10 total score		WHO-5 total score	
*Background characteristics*	*Mean (SD)*	*range*	*Mean (SD)*	*range*
*Sex*				
Female	24.0 (5.5)	7–40	15.4 (3.8)	2–25
Male	24.2 (6.3)	7–40	15.7 (4.2)	0–25
*p*-value	0.717		0.547	
*Programme*				
Undergraduate	23.8 (5.9)	7–40	15.3 (3.9)	2–25
Postgraduate	24.9 (5.3)	10–40	15.8 (4.0)	0–25
*p*-value	0.020		0.131	
*Senior year student*				
No	24.1 (6.0)	7–40	15.1 (4.0)	0–25
Yes	24.1 (5.4)	8–40	16.0 (3.8)	4–25
*p*-value	0.912		0.003	

*p*-values are computed by independent *t*-test

### Perceived well-being

The total scores in WHO-5 ranged from 0.0 to 25.0, with a mean of 15.5 (SD = 3.9). Perceived well-being among undergraduates and postgraduates was not significantly different (*p* = .131), but there was a statistically significant difference between junior and senior students (*p* = .003). Seniors, in their 4th or 5th years as undergraduates and in their 2nd or 3rd years as postgraduates, reported higher scores in WHO-5, reflecting a better perceived level of well-being.

### Predictors of perceived well-being

The results of the bivariate analyses and multivariable regression analysis of perceived well-being among university nursing students in Hong Kong are shown in Table 3. Only the year of study and self-reported resilience showed significant bivariate associations with perceived well-being. Self-reported resilience had a medium, positive correlation with perceived well-being (*r* = .378, *p* < .001) and senior students had significantly higher level of perceived well-being than junior students (16.0 vs 15.1, *p* = .003). Furthermore, the multivariable regression analysis revealed that both self-reported resilience (regression coefficient B = 0.259; *p* < .001) and the year of study (senior vs junior, B = 0.875; *p* = .002) were significantly and independently associated with perceived well-being and collectively explained 15.5% of the variance.

**Table 3:** Factors associated with perceived well-being (WHO-5 total score) in the university nursing students

	Bivariate analysis^a^		Multivariable analysis^b^		
	Mean (SD)/correlation coefficient	P	B	SE	P
*Background characteristics*					
*Sex*					
Female (ref)	15.4 (3.8)	0.547	NE		
Male	15.7 (4.2)				
*Programme*					
Undergraduate (ref)	15.3 (3.9)	0.131	NE		
Postgraduate	15.8 (4.0)				
*Senior year student*					
No (ref)	15.1 (4.0)	0.003			
Yes	16.0 (3.8)		0.875	0.279	0.002
*Self-reported resilience*					
CD-RISC-10 total score	0.378	<0.001	0.259	0.024	<0.001
			R^2^ = 0.155		

^a^Pearson’s correlation coefficients between the independent variables and the outcome are presented for continuous independent variables, whereas mean (standard deviation) of the outcome variable are presented for categorical independent variables

^b^Only those examined independent variables with *p* values <0.05 in bivariate analyses were selected for multivariable regression analysis

NE: not being entered into multivariable regression

B: regression coefficient

SE: standard error of the regression coefficient

ref: reference group of the independent categorical variables

R^2^: proportion of variance explained by the multivariable regression model

## Discussion

The results support the hypothesis that nursing students with a high level of resilience have better perceived well-being. The results also show that the level of resilience of postgraduates was significantly higher than that of undergraduates. The perceived well-being of senior students was significantly better than that of junior students.

### Resilience

The study found that the resilience level of Hong Kong university nursing students was generally lower than that of their counterparts in other countries [11, 12], a result that may be attributed to the stressful study and clinical environment in Hong Kong. A survey of stress and burnout among Hong Kong nursing students reported an increased level of stress, burnout, and psychological morbidity across semesters [27]. The perceived stress level correlates negatively with resilience [28]. The present result suggests a supportive learning environment is needed to build up resilience among Hong Kong university nursing students. The postgraduate students in this study were more resilient than undergraduate students. Ample evidence shows that resilience is developed through a process where people acquire resilient qualities by overcoming adversity [4, 29, 30]. The high level of resilience in postgraduate students reflects their maturity and personal growth through various vicissitudes. The successful experience of completing their undergraduate degree may help them develop coping skills to withstand hardships and meet the demands of postgraduate study.

There was no significant difference in resilience level between junior and senior students. The result is consistent with a study on Australia nursing students, whose resilience level did not significantly change during their nursing studies [31]. A study that used the grounded theory to examine the acquisition of resilience in nursing students reveals that they experience a transient disengagement process before realising the growth that has resulted from passing through adversity and becoming ready for the next challenge [32]. They are exhausted from continual adversity during this process. Both junior and senior students in a nursing programme have to deal with different stressors. The former focus on meeting the demands of a new programme, the academic workload and clinical environment. Although the latter deal with these challenges and move on to their last year of study, they are concerned with developing into competent graduate nurses. The continual tension experienced by senior students may deter them from significantly improving their resilience level.

### Well-being

Overall, students reported a medium level of perceived well-being. Multivariable regression further found that the year of study was a significant predictor of perceived well-being, with senior-year students showing better perceived well-being - which is contrary to the findings of previous studies. One longitudinal study showed that the psychological well-being of university students progressively worsened from year one to year three [33]. Smith and Yang’s cross-sectional study found a similar trend, in that the psychological well-being of Chinese nursing students was better in their junior than in their senior years [34]. They explained that senior students suffered great stress from academic study and clinical practicum. To our knowledge, the clinical practicum of most nursing programmes in mainland China is arranged in the final year of study. The clinical practicum is identified as a significant stressor for nursing students, particularly those unprepared to manage multiple demands from the real clinical environment [4, 30]. Our cohort of nursing students starts their clinical placement in the junior year of study. An empowerment workshop that prepares students to meet the learning outcomes of clinical placements is arranged for each year of study. Early exposure and preparation for clinical placement probably helps students to adapt to the clinical environment and to improve their personal well-being. Compared with students in Smith and Yang’s study [34], our cohort consists of higher percentage of male students (25.1% vs 2.7%). Although our study results do not show a significant difference in perceived well-being between genders, the student profile is comparable to the global trend of increasing males entering the nursing profession [35].

### Relationship between resilience and well-being

The results of the present study show that there is a medium and positive relationship between resilience and well-being among university nursing students. Further, resilience is a significant predictor of perceived well-being in this group of students. Our finding supports the association between resilience and personal well-being that was found in previous studies. Bore et al. reported that high resilience levels significantly predict better psychological well-being in undergraduates studying psychology [36]. A medium positive correlation between resilience and psychological well-being was also demonstrated by Zeng, Hou, and Peng in Chinese primary and secondary school pupils [37]. They reported that resilience mediates between psychological well-being and a grown mindset, which pertains to the ability of an individual to regard challenges as opportunities for intellectual growth [37]. Resilient people can identify positive meanings in adversity that buffer their negative emotions [14, 38]. Such adaptive emotion regulation is associated with improved well-being [39]. Positive thinking should therefore be emphasised in nursing education because it plays a significant role in mediating resilience and well-being.

### Limitations

The study has several limitations. As it is a cross-sectional study causality between resilience and perceived well-being cannot be established. We are also unable to observe individual changes in these variables across years of study. Future research may use a prospective, longitudinal design to reveal the trends, with results perhaps susceptible to socially desirable response bias because the variables are measured with self-report instruments [40]. In addition, the understanding between resilience and perceived well-being is restricted by the limited demographic data collection. More personal information should be collected in future studies, such as learning environment and psychosocial background including household, number of siblings, religious beliefs, whether in a relationship, previous work experience, emotional intelligence and happiness index.

### Implications

The present study shows the resilience level of Hong Kong university nursing students to be relatively low and stagnant. The significant correlation between resilience and perceived well-being suggests directions for improving matters in these areas. The results highlight the significance of curriculum design and a supportive learning environment in promoting the resilience and well-being of nursing students and reducing their vulnerability [41]. Clinical placements that commence in the junior year assist nursing students to adapt to a demanding clinical environment and to develop their clinical competence. This arrangement minimises the shock of reality and stress from clinical practice and possibly enhances their personal well-being. In addition, mentorship programmes and skills revision workshops for junior-year students before the start of clinical practice probably helps them to prepare better for the challenges and provides additional support. The effect of such clinical arrangement on student learning and well-being should be examined in future studies. The curriculum should include learning modalities that strengthen students’ resilience to cope with various stressors and possible adversity [13]. For instance, problem-based learning develops problem-solving skills, critical thinking and persistence, which build up students’ resilience [42]. Reflective writing provides students with the opportunity to analyse issues from different perspectives and identify positive meanings from experience, learning to maintain positive attitudes during hardship by means of such exercises. Effective coping strategies and emotion regulation are psychosocial skills that can be incorporated into the curriculum to strengthen resilience [4, 43]. A student-centered learning environment with positive expectations towards students is also desirable to resilience building [26].

In addition, the present study identifies higher level of resilience in postgraduate students and establishes the association between resilience and perceived well-being in nursing students. Future research should explore the mediating factors for such relationships, and causal relationship between these outcome variables. The findings may have implications to future curriculum development.

## Conclusions

Nursing education focuses not only on knowledge of nursing techniques and clinical skills, but also on developing personal qualities. This study demonstrates that there is a significant relationship between resilience and well-being in nursing students, and the results suggest a need to strengthen their resilience to improve personal well-being, and improve perceived well-being to build up their resilience. Approaches to developing this personal quality and well-being should be integrated into a nursing curriculum. A robust development of resilience is imperative to students’ survival in nursing studies and their future professional careers.

## Abbreviations

CD-RISC-10: 10-item Connor-Davidson Resilience Scale
SBREC: Survey and Behavioural Research Ethics Committee
SPSS: Statistical Package for the Social Sciences
WHO-5: World Health Organisation-5 Well-Being Index
